# Cross-serotype interactions and disease outcome prediction of dengue infections in Vietnam

**DOI:** 10.1038/s41598-019-45816-6

**Published:** 2019-06-28

**Authors:** R. Aguas, I. Dorigatti, L. Coudeville, C. Luxemburger, N. M. Ferguson

**Affiliations:** 1 MRC Centre for Global Infectious Disease Analysis, School of Public Health, Imperial College London, Faculty of Medicine, Norfolk Place, London, W2 1PG UK; 2 Sanofi Pasteur, 14 espace Henry Vallée, 69007 Lyon, France; 3 Centre for Tropical Medicine and Global Health, Nuffield Department of Medicine, University of Oxford, Oxford, United Kingdom; 4 Mahidol-Oxford Tropical Medicine Research Unit, Faculty of Tropical Medicine, Mahidol University, Bangkok, Thailand

**Keywords:** Viral infection, Applied mathematics

## Abstract

Dengue pathogenesis is extremely complex. Dengue infections are thought to induce life-long immunity from homologous challenges as well as a multi-factorial heterologous risk enhancement. Here, we use the data collected from a prospective cohort study of dengue infections in schoolchildren in Vietnam to disentangle how serotype interactions modulate clinical disease risk in the year following serum collection. We use multinomial logistic regression to correlate the yearly neutralizing antibody measurements obtained with each infecting serotype in all dengue clinical cases collected over the course of 6 years (2004–2009). This allowed us to extrapolate a fully discretised matrix of serotype interactions, revealing clear signals of increased risk of clinical illness in individuals primed with a previous dengue infection. The sequences of infections which produced a higher risk of dengue fever upon secondary infection are: DEN1 followed by DEN2; DEN1 followed by DEN4; DEN2 followed by DEN3; and DEN4 followed by DEN3. We also used this longitudinal data to train a machine learning algorithm on antibody titre differences between consecutive years to unveil asymptomatic dengue infections and estimate asymptomatic infection to clinical case ratios over time, allowing for a better characterisation of the population’s past exposure to different serotypes.

## Introduction

Dengue infections are the most common vector borne viral infections worldwide, with approximately one half of the world population at risk of acquiring a dengue infection^1^, with 390 million new infections estimated to occur in tropical and subtropical countries each year, of which one fourth to one third are clinical^2,3^. The dengue virus (DENV) is a single-stranded, positive-sense RNA virus of the *Flavivirus* genus that exists in the form of 4 distinct (but closely related) serotypes – DENV1 to DENV4. All serotypes co-circulate in hyperendemic areas causing periodic acute epidemics with significant occurrence of dengue fever and dengue haemorrhagic fever cases, with cyclical replacement of the dominant serotype over time^4,5^. These oscillatory dynamics have been postulated to be driven by cross-protection across serotypes and/or enhancement of infection by heterologous DENV strains^4,6–9^. Indeed, whilst there is certainly host and viral factor modulation of disease severity^10–17^, the host immune landscape stands as the key risk determinant of a dengue infection’s clinical outcome^18–22^.

In high transmission intensity settings, most individuals experience more than one infection, thus making immune interactions between the cyclically dominant serotypes extremely relevant. Classical human challenge studies^19^, now replicated in non-human primates^23^, reveal transient protection from dengue disease and/or high viral load upon heterologous challenge in individuals primed with a specific viral serotype. What follows this short-lived heterologous immunity has been the focus of much scrutiny with a risk enhancement phenomenon emerging as a result of either antibody dependent enhancement^24,25^ or immunopathogenesis akin to the original antigenic sin^26,27^. Exposure to a dengue infection induces a strong homologous immunity which was previously thought to be complete and lifelong^28^, but now understood to be only partial^29,30^ and age dependent^21,31^. Subsequent exposure to a heterologous DENV serotype has been suggested to increase the risk of a clinical dengue outcome, resulting in higher rates of and dengue haemorrhagic fever in secondary infections^22,32,33^. Whilst some qualitative estimates of the generic strength and duration of cross-immunity have informed models fitted to epidemiological data^6,7,34^, it is still unclear whether pre-exposure to specific serotypes protects against or enhances the probability of illness upon subsequent infections with specific heterologous serotypes.

Longitudinal data in a high incidence setting where all 4 serotypes co-circulate would be ideal to understand how different immunity profiles modulate the clinical outcome of dengue infections. Several prospective cohort studies have provided valuable insight into immunity-related pathogenicity trends, decisively demonstrating that antibody dependent disease enhancing effects do occur in a non-serotype specific manner^35–38^, showing how the risk of clinical illness is modulated by a rapidly changing antibody repertoire^3^, identifying serological markers of secondary dengue infections for improved clinical management^39–41^, and suggesting a complex interaction between viral genetics and population dynamics of serotype-specific immunity in determining clinical outcomes^17^. However, no study has been able to disentangle serotype specific enhancement interactions, showing how pre-exposure antibody levels against a specific serotype modulate the likelihood of clinical illness with another serotype. A recent prospective cohort study of dengue infections in schoolchildren in Vietnam^42^ collected detailed clinical and immunological data over the course of 6 years (2004–2009). The study reported 60% of infections to be secondary but found that the hospitalisation rate was similar for primary and secondary infections and the proportion of severe cases was similar in primary and secondary hospitalised cases. Cases were defined as primary or secondary given the acute sera IgM/IgG titre ratios, but used a threshold of 1.8 which was likely too large^39,40,43^. In that study, routine blood collection at the beginning of the year was also done for a sample of the cohort, allowing us to investigate two main issues here: (1) the serotype specific risk of clinical illness in individuals with different previous dengue virus exposures; (2) whether neutralizing antibody levels measured by the Plaque Reduction Neutralization Test (PRNT) would be a reliable serotype specific indicator of previous dengue exposure.

We then explore correlations between immunological baseline scenarios (using routinely collected sera) and future clinical outcomes, offering a comprehensive picture of dengue serotype interactions and highlighting how knowledge of the current antibody repertoire of an individual can help predict the severity of a potential future infection with the currently circulating serotype(s). This can be particularly important when considering deploying dengue vaccines. We also demonstrate how antibody titre differences between consecutive years can be used to accurately predict dengue asymptomatic infections, revealing the complex dynamical nature of the asymptomatic infection to clinical dengue case ratio.

## Materials and Methods

### Data

Full details on the clinical study, methodology, assays used and data collection have been published in^42^. In summary, school aged children from a population of 249,535 inhabitants were enrolled into the study at three nursery schools, two primary schools, and one secondary school in southern Vietnam. This was a dynamic cohort with recruitment onto the lowest age group and replacement of drop-outs into the respective age classes. Enrolled children were actively and passively monitored year-round for febrile episodes. Absentees were visited at home by study nurses and children with an axillary temperature ≥38 °C were taken to the paediatric ward. During the school holiday trimester all children were visited at home three times a week. All hospitalisations were documented, whatever the reason. This study was approved by the scientific committee of the Pasteur Institute Ho Chi Minh City and by the Ministry of Health of Viet Nam. Written informed consent was obtained from the parents or legal representatives of all participants prior to enrolment. All experiments were performed in accordance with relevant guidelines and regulations.

Clinically-suspected dengue cases were defined as acute febrile illness events (reported by the adult caretaker or confirmed by axillary temperature ≥38 °C lasting over 2 days) for which a diagnosis of dengue or viral infection (i.e. acute fever without clinical signs of focused infection) was suspected. Two blood samples were collected for laboratory confirmation of each suspected dengue case: the first (acute sera) at consultation and the second (convalescent) either upon discharge of hospitalised cases or 10 days after outpatient consultation. Dengue infections were confirmed by one of the following virological assays: virus isolation, NS1 antigen assay and quantitative real-time polymerase chain reaction (qRT-PCR). Throughout this paper we refer to clinical dengue illness as symptomatic dengue infections with virological confirmation by virus isolation, NS1 or qRT-PCR assay and define asymptomatic infections as presumptive dengue infections without any apparent symptomatology.

Seroprevalence surveys were performed each December (low transmission season), at which time blood samples were collected from all enrolled children to assess the presence of antibodies (via ELISA), along with anthropometric and vital signs measurements. In a randomized subset of 200 subjects from the universe of enrolled children, serotype-specific quantification of the titre of neutralising antibodies (PRNT50) were performed. Due to this annual randomisation, consecutive PRNT50 titre measurements for the same individuals are infrequent. Sera from all dengue laboratory-confirmed cases were retrospectively analysed by PRNT50, thus providing a December snapshot of the antibody profiles of children later developing dengue illness. The total pool of analysed PRNT50 samples is then the aggregate set of random annual samples and all children that had confirmed dengue fever.

### Statistical analyses

#### Overview

Due to the study design, individual serological follow-up was intermittent, with PRNT50 measurements restricted to annual surveys and clinical cases. The same individuals were not necessarily (and indeed infrequently) surveyed on consecutive years, thus we opted to analyse each person-year separately and investigate the variables affecting the risk of clinical dengue during each follow-up year. Only records for people with measured antibody titres and a known clinical endpoint at the end of the year were included.

We use the longitudinal data collected in^42^ to address two main questions, each involving the use of different analytical techniques as discretised below: *(i)* how antibody titres measured at the beginning of each year modulate the serotype-specific risk of clinical illness and *(ii)* whether PRNT50 antibody titres can be used as a reliable serotype-specific indicator of previous dengue exposure.

### How antibody titres modulate the serotype-specific risk of clinical illness

A baseline immunity effect was estimated by analysing the relative risk of clinical dengue illness in individuals with a different immune status (as quantified by PRNT50) at the beginning of each year (baseline). The relative risk of clinical dengue illness is the ratio between the clinical dengue illness rates in immune and naïve individuals, calculated as: OR = PI/PN, where PN and PI are the observed clinical dengue illness occurrence odds in naïve and immune individuals over each year, respectively. PI was calculated by dividing the number of clinical dengue illness cases (C) by the number of non-cases (NC) for each baseline immunity status. In a first instance we conservatively considered individuals with a PRNT50 titre below the detection threshold (<1/10 dil) to be immunologically naïve (this assumption is relaxed later) and consider the effects of serotype transcendent immunity (i.e. against any dengue serotype). We later calculated serotype-specific odds ratios clinical dengue illness, by accounting for serotype-specific cases in the numerator and excluding any case (i.e. caused by any serotype) from the denominator. For instance, the odds ratio of clinical dengue illness with serotype *i* was calculated by dividing the number of clinical dengue illness cases caused by serotype *i* by the number of non-cases (i.e. also excluding individuals infected with serotype *j*≠*i*) for each baseline immunity status. For each reference serotype, PRNT50 titres were scanned and individuals were clustered into bins according to the measured homologous titre and the highest titre recorded among the heterologous serotypes. We used 6 bins, classifying titres into the following intervals: <10; 10–19; 20–39; 40–79; 80–159; ≥160. These intervals reflect the nature of the assay, where two-fold dilutions are performed until the number of plaque forming units is reduced by 50% compared to the serum free counterfactual. It is reported with a minimum value of 5 if no neutralisation occurs, 10 if one dilution is performed, 20 for two dilutions, and so on. If both measured homologous and heterologous titres fall in the first bin, the individual was considered fully naive. We then calculate the odds ratio of having clinical dengue illness with a specific serotype for PRNT50 titre bin *k* as:

$$O{R}_{i,k\ne 1}=\frac{{C}_{i,k\ne 1}/N{C}_{k\ne 1}}{{C}_{i,k=1}/N{C}_{k=1}}$$

where k = 1 represents fully naïve children. We used multinomial logistic regression to determine the association between the occurrence of clinical dengue illness and a set of independent variables describing the immunological status of each child including PRNT50 titres against DENV1–4 and IgG and IgM measurements (see Supplementary Table S2), as well as ag, gender, and calendar year.

Several immunity descriptive summary variables were created to reflect the breadth of individual antibody profiles as summarised in Supplementary Table S2. We evaluated different models containing different combinations of predictors (Supplementary Table S3) using the Akaike Information Criterion (AIC), which is defined as $AIC=2{n}_{p}-2\,\mathrm{ln}(L)$, where *n*_*p*_ is the number of estimated parameters and *L* denotes the maximized likelihood. The predictive performance of each model was also assessed by calculating the area under the receiver operating characteristic (ROC) curve.

### Multinomial logistic regression

Logistic regression methods are valuable tools to assess the effects of multiple explanatory predictors (numeric and/or categorical) on an outcome variable. A method of this kind was used to differentiate primary from secondary dengue infections, as defined by convalescent PRNT titres, using only acute serology results in^40^. When dealing with a polytomous outcome variable, one can use a natural extension of the binary logit model, the multinomial logistic model. It explains the relative risk of having a specific outcome versus a reference category, *k*, using a linear combination of *p* predictor variables. It can be written as:

$$\mathrm{ln}(\frac{{\pi }_{i}}{{\pi }_{J}})={\alpha }_{i}+\sum ^{p}{\beta }_{ip}{X}_{p}$$

where *π*_*i*_ is the probability outcome *i, J* is the number of response categories (in this case, all possible clinical outcomes, including non-cases), α is the intercept, and *p* is the number of predictors. Labelling non-cases as the *J*^*th*^ outcome category, we solve a total of 4 equations simultaneously (one for each serotype) to estimate the coefficients *β*. Assuming the coefficients for the non-cases to be zero, the probability of having dengue clinical illness with serotype *j* is given by:

$${\pi }_{j}=\frac{{e}^{{\alpha }_{j}+\mathop{\Sigma }\limits^{p}{\beta }_{jp}{X}_{p}}}{1+\sum _{j=1}^{J-1}{e}^{{\alpha }_{j}+\mathop{\Sigma }\limits^{p}{\beta }_{jp}{X}_{p}}},j=1,\mathrm{...},J-1$$

The log likelihood of the multinomial logistic regression model can then be expressed as^44,45^:

$$l(\beta )=\sum _{i=1}^{N}\sum _{j=1}^{J-1}({y}_{ij}\sum _{k=0}^{K}{\beta }_{kj}{X}_{ik})-{n}_{i}\,log(1+\sum _{j=1}^{J-1}{e}^{{\sum }_{k=0}^{K}{\beta }_{kj}{X}_{ik}})$$

where *y*_*ij*_ contains the observed counts of the *j*^*th*^ outcome variable in *n*_*i*_ children. We use an iteratively weighted least squares algorithm available in the statistical toolbox of Matlab 2.14 to find the maximum likelihood estimates.

This framework provides an intuitive interpretation of the estimated coefficients. Taking coefficient *β*_11_ as an example, it indicates how many times – exp(*β*_11_) – the probability of a child having a clinical DENV1 illness increases for each unit increase in anti-DENV1 antibody titre (all else being constant). Negative coefficients thus reflect a decrease in likelihood of disease with increasing antibody titres. If we use a log2 transformation of the measured PRNT50 titres, the estimated coefficients *β* then reflect the relative probability of dengue illness for each additional log2 dilution.

### The use of PRNT50 antibody titres as serotype-specific indicators of dengue exposure

We used machine learning techniques to analyse the occurrence of asymptomatic dengue infections. Similar techniques have been used recently to impute immunological status^46^ and identifying subclinical infections^3^. Using a subset of our longitudinal data limited to individuals that had antibody titre measurements taken in two consecutive years, we trained a random forest algorithm (RFA) on individuals known to have been cases and those displaying no change or a decrease in antibody titres from one year to the next. This comprised a set of 363 individuals. We then used the resulting random forest model to predict the outcome of all others, i.e., children with an antibody rise (of any magnitude) for at least one serotype. It should be noted that the training set was comprised of clinical cases only, thus the predicted infections should have comparable antibody titre rises to those observed in clinical cases. A subset of the training data mentioned above was used to develop a serotype-specific RFA to predict the infecting serotype for the predicted non-clinical infections. Six un-typed clinical infections were not used to inform this RFA and were instead included in the subset of infections to predict. We can then easily calculate the proportion of children with sequential PRNT50 measurements which are predicted to have been infected by dividing the number of predicted asymptomatic infections by the number of individuals with sequential PRNT50 measurements. Assuming the remaining children in the population experience the same annual risk of exposure, we can extrapolate the number of expected sub-clinical infections in the whole study cohort. To calculate the asymptomatic infection to clinical case ratio we divide the number of predicted number asymptomatic infections in the whole cohort by the number of observed clinical dengue cases.

### Random forest algorithm

The random forest algorithm (RFA) is an ensemble classifier with multiple low correlation decision trees aggregating into a low bias and low variance “forest”. Each tree in a random forest is trained on a bootstrap of the data, and each tree branch contains a random subset of all available variables. Final classification of each sample results from aggregating the votes of all trees in the forest. This algorithm has proven to be extremely accurate in classification tasks^47^, partly due to the embedding of the feature onto the model training process, and the integral usage of the training data (whereas other methods require a split into training and validation sets)^48^. Crucially, the classifier’s performance is evaluated on out-of-bag samples (samples left out of the bootstrapped data for each tree) in the form of a misclassification error. Variable importance is also estimated from the samples which are left out of the training set at each split of the tree, making the random forest algorithm very robust to over-fitting. In fact, random forests have been demonstrated not to over-fit as the number of trees grows to infinity, instead producing a limiting value of the prediction error^47^. We used the RandomForest R package to implement the RFA.

## Results

### Epidemiological trends

Children were enrolled into the study from December 2003 until December 2006 as displayed in Table 1, with the cohort size peaking in 2005 and the mean age consistently increasing as a consequence. No additional children were recruited in December 2007 and December 2008 in order to decrease the workload of the investigation team.

**Table 1:** Cohort characteristics and serology subsets at the beginning of each follow up year.

	2004	2005	2006	2007	2008	2009
	N (%)	N (%)	N (%)	N (%)	N (%)	N (%)
Number of enrollees	2190	1645	722	652	—	—
Size of the cohort in Jan.	2190	3239	3146	3081	2350	1775
**Sex**						
Male	1103 (50.4)	1593 (49.2)	1559 (49.6)	1551 (50.3)	1207 (51.4)	908 (51.2)
Female	1087 (49.6)	1646 (50.8)	1587 (50.4)	1530 (49.7)	1143 (48.6)	867 (48.8)
Age (years)						
Mean +/− SD	6.89 +/− 2.11	8.45 +/− 278	8.53 +/− 2.87	8.55 +/− 2.91	9.13 +/− 2.59	9.66 +/− 2.24
Min; Max	3; 10	3; 14	2; 15	3; 15	4; 15	5; 15
Median	7	9	9	9	9	9
**Serology**						
Number of PRNT50 measurements from sera collected in the annual surveys	259	270	92	296	148	138
Clinical cases in the year following those PRNT50 measurements	53 (0.205)	39 (0.144)	78 (0.848)	113 (0.382)	9 (0.061)	28 (0.203)

Incidence of dengue illness in the serology subset displayed a similar age profile for both genders (Supplementary Fig. S1). An interesting serotype replacement pattern emerges, with DENV2 being progressively replaced by DENV1 (Supplementary Fig. S1). Both DENV3 and DENV4 represent a much smaller proportion of all sampled viruses and do not exhibit any significant temporal trend.

### PRNT50 titres as indicators of immune protection/enhancement

The distributions of PRNT50 titres against each serotype are skewed, with the bulk of individuals presenting with very low titres (58% of individuals having no detectable neutralizing antibodies and 70% presenting with PRNT50 < 20 against all serotypes). This means that standard boxplots would not be a good representation, thus we opted to display the density distributions of antibody titres through violin plots (Supplementary Fig. S2 – right side panels). Pre-infection homologous antibody titres in individuals presenting with a clinical dengue illness caused by the same serotype are consistently low. Strikingly, individuals presenting with clinical dengue illness caused by DENV1 and DENV3 had similar antibody titre profiles, with high anti-DENV2 titres (means of 91.3 and 93.1 respectively), lower anti-DENV3 and anti-DENV4 titres, and very low to undetectable anti-DENV1 antibodies (means of 11.6 and 7.4 respectively). Also noteworthy are the high anti-DENV1 and anti-DENV2 antibodies (means of 146.9 and 85.8 respectively) found in DENV4 cases, as well as the high anti-DENV2 and anti-DENV4 antibodies in cases infected by DENV3 (means of 93.1 and 61.8 respectively). DENV1-infected cases had high anti-DENV2 levels (mean PRNT50 = 92), whereas DENV2 cases displayed the highest anti-DENV3 antibody titres (mean PRNT50 of 74.0).

Children with detectable neutralizing antibodies to any of the 4 serotypes were less likely to become clinically ill (regardless of infecting serotype) within the year following the blood sample, compared to completely naïve children (OR = 0.752 (0.577–0.979); p = 0.0346). Considering individuals with a PRNT50 titre over 80 against any serotype as immune leads to an even lower and more statistically significant relative risk (OR = 0.643(0.483–0.856); p = 0.0025) (Supplementary Table S1). Discretising immune profiles further, we classified children into immunity level categories for each serotype, according to their measured titre for both homologous and heterologous titres (Table 2). Children with a combination of low homologous titres (PRNT50 < 40) and intermediate-to-high heterologous titres (PRNT50 ≥ 40) have increased odds of having a dengue clinical episode relative to fully naïve individuals. Conversely, immune individuals to both homologous and heterologous serotypes (PRNT50 > 40) are protected against disease.

**Table 2:** Odds ratio (OR) of dengue illness with a specific serotype according to the immune status at the beggining of each year.

**Serotype 1**							
	**Heterologous titres**						
Homologous titre		<10	[10,20[	[20,40[	[40,80[	[80,160[	≥160
	<10	1.00	^3^ 3.05^*^	^3^ 1.36	^3^ 1.05	^3^ 0.87	^3^ 1.32
	[10,20[	0.00	0.00	0.00	0.00	^2^ 1.38	^3^ 0.50
	[20,40[	0.00	0.00	0.00	0.00	^2^ 3.62	^3^ 0.89
	[40,80[	0.00	0.00	0.00	0.00	0.00	^3^ 0.52
	[80,160[	0.00	^2^ 0.00	0.00	0.00	0.00	^3^ 0.49
	≥160	0.00	^2^ 0.00	^3^ 0.66	^2^ 0.00	^2^ 0.00	^3^ 0.13^*^
**Serotype 2**							
	**Heterologous titres**						
Homologous titre		<10	[10,20[	[20,40[	[40,80[	[80,160[	≥160
	<10	1.00	^2^ 4.28^+^	^3^ 0.51	^3^ 1.22	^3^ 2.57	^3^ 2.57
	[10,20[	0.00	38.06^*^	0.00	^2^ 5.77^*^	^2^ 5.77^*^	^3^ 1.97
	[20,40[	0.00	0.00	0.00	^2^ 4.23	^2^ 0.00	^3^ 1.81
	[40,80[	^2^ 0.00	0.00	0.00	0.00	0.00	^3^ 0.75
	[80,160[	^3^ 0.00	^3^ 0.00	0.00	0.00	0.00	^3^ 2.87^+^
	≥160	^3^ 0.00	^3^ 0.00	^3^ 0.00	^3^ 0.00	^2^ 0.00	^3^ 0.00
**Serotype 3**							
	**Heterologous titres**						
Homologous titre		<10	[10,20[	[20,40[	[40,80[	[80,160[	≥160
	<10	1.00	^2^ 2.71	^3^ 2.24	^3^ 1.20	^3^ 2.85	^3^ 1.47
	[10,20[	0.00	0.00	^2^ 0.00	^2^ 9.07^*^	^3^ 4.98^*^	^3^ 3.96^+^
	[20,40[	0.00	0.00	0.00	0.00	^2^ 0.00	^3^ 3.96^*^
	[40,80[	0.00	0.00	0.00	0.00	0.00	^3^ 2.80
	[80,160[	0.00	0.00	0.00	0.00	0.00	^3^ 0.00
	≥160	0.00	^2^ 0.00	3 0.00	^3^ 0.00	^2^ 0.00	^3^ 0.00
**Serotype 4**							
	**Heterologous titres**						
Homologous titre		<10	[10,20[	[20,40[	[40,80[	[80,160[	≥160
	<10	1.00	4.34	^2^ 2.30	^3^ 5.42^*^	^3^ 0.50	^3^ 1.89
	[10,20[	0.00	0.00	0.00	0.00	0.00	^3^ 1.45
	[20,40[	^2^ 0.00	0.00	0.00	0.00	39.08^*^	^3^ 0.00
	[40,80[	^3^ 0.00	^2^ 0.00	0.00	0.00	0.00	^3^ 3.35
	[80,160[	0.00	^2^ 0.00	0.00	0.00	0.00	^3^ 0.00
	≥160	^2^ 0.00	0.00	^2^ 0.00	0.00	0.00	^3^ 0.00

The heterologous titres refer to the maximum PRNT50 titre measured across the non-homologous titres (e.g. for serotype 1, the heterologous titre is the maximum of the PRNT50 titres against DENV2–4). The numbers on the top left corner of each cell represent the degree of evidence, where the degree is equal to 1 if there are insuficient data to extrapolate any meaningful coeficients (sum of cases and non-cases lower than 5). Degree values of 1 are omitted here for clarity. The degree of evidence is equal to 2 when the sum of cases and non-cases between 5 and 10 and translates to estimated coefficients with not enough data for significance. Degree values of 3 indicates coefficients for which there were enough data for statistical significance (sum of cases and non-cases greater than 10).

^+^significant at p < 0.1; ^*^significant at p < 0.05; ^**^significant at p < 0.01

### Cross-serotype interactions

To disentangle the interactions between antibody titre measurements and other factors such as age and gender in determining the likelihood of a given clinical outcome, we undertook an exhaustive multinomial model selection process that explored the predictive power of several covariates (Tables S2, S3). Ultimately, the most parsimonious model (as per AIC) that accurately fits the observed dengue clinical outcomes is one informed on baseline PRNT50 titres against each of the serotypes, age and year of sample collection (see Supplementary Table S4). Summary measures of immunity describing the richness of the immune profile (such as dominance or skewedness) and demographic indices (age, gender) do not improve the model fit significantly. The estimated coefficients obtained with model 49 are shown in Table 3. As expected, estimated homologous serotype coefficients are negative in all models, meaning having higher titres against one serotype decreases the odds of having dengue illness with that same serotype, although this effect is only significant for DENV1 and DENV2. The homologous antibody effects in DENV3 and DENV4 displayed the largest negative coefficients, suggesting a strong protective effect, but were ultimately not statistically significant due to the small number of cases infected with those serotypes. A detailed exploration of the homologous titres protective effect is given in Supplementary Table S9.

**Table 3:** Estimated coefficients for the most likely model (measured by AIC).

	Dengue 1	Dengue 2	Dengue 3	Dengue 4
Intercept	2.229^+^	0.218	20.893	5.192
*X1*	−0.719^**^	0.292*	−3.757	0.360^*^
*X2*	0.001	−1.076^**^	0.311^+^	0.088
*X3*	−0.107	0.033	−1.889	−0.341
*X4*	−0.028	0.247	0.656^**^	−1.997
*Age*	−0.077^+^	0.159*	−0.069	0.136
*Year*	0.404^**^	−0.547^**^	0.187	−0.017

^+^significant at p < 0.1; ^*^significant at p < 0.5; ^**^significant at p < 0.01.

We also uncover a series of cross-serotype interactions manifested in the risk of acquisition of dengue illness as follows:

- Clinical DENV1 illness: no significant effect of PRNT50 titres against any of the other serotypes on the odds of clinical DENV1 illness detected.
- Clinical DENV2 illness: we found significantly increased odds of clinical DENV2 illness in children with high titres against DENV1. Supplementary Fig. S2 shows that several children with high antibody titres against DENV1 at the time of the annual sero-surveys succumbed to DENV2 or DENV4 illness (top right panel).
- Clinical DENV3 illness: we found that elevated titres against DENV1 confers protection against clinical DENV3 illness and that the presence of anti-DENV2 or anti-DENV4 titres significantly enhance the odds of clinical DENV3 illness.

- Clinical DENV4 illness: high anti-DENV1 titres increase the odds of clinical DENV4 illness.

**Figure 1:**
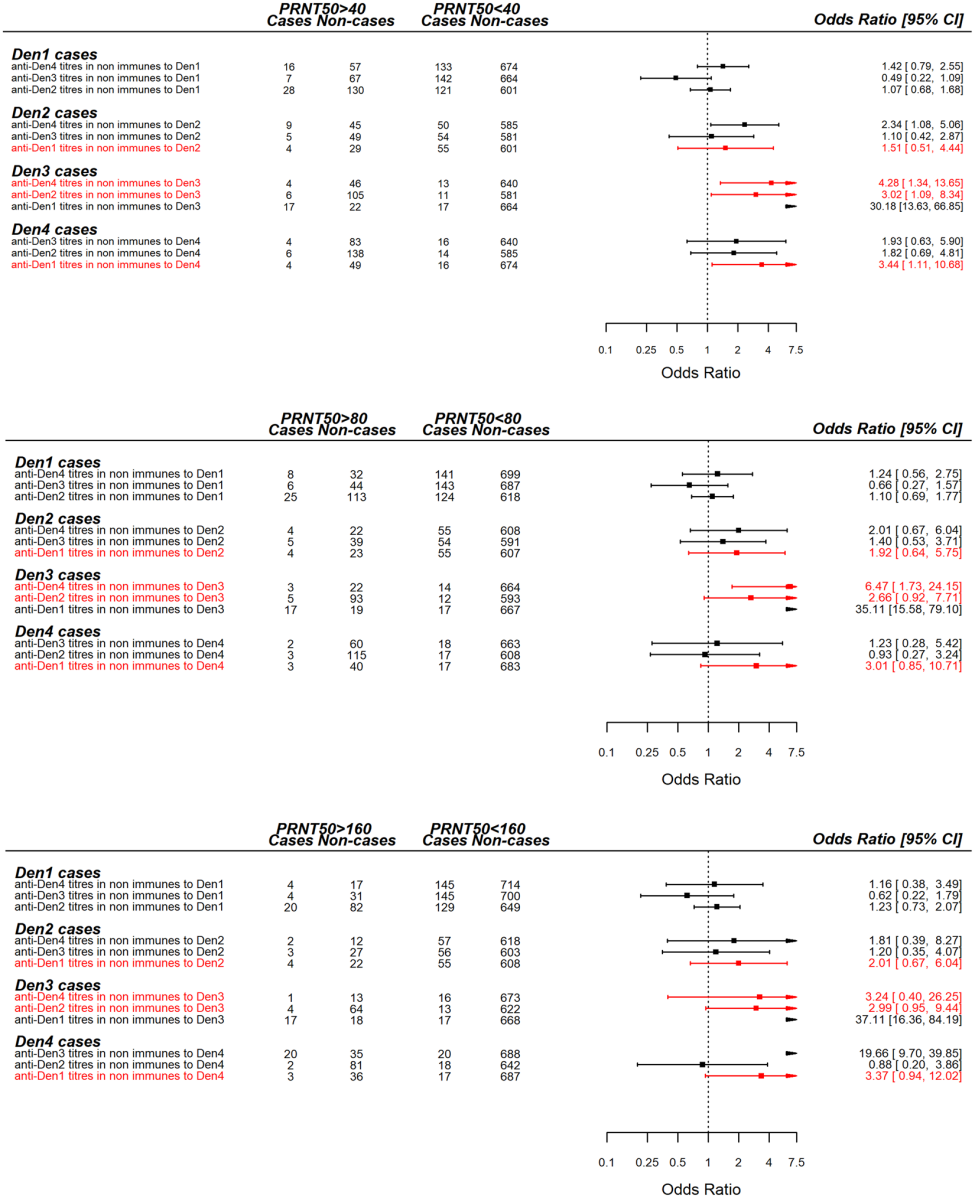
Odds ratio of dengue illness in the year following neutralising antibody titre measurements. The odds ratio presented here refers to children with a homologous PRNT50 titre under 40, i.e., non-immune to the reference dengue serotype thus reflecting the ratio between the odds of having clinical dengue illness when immune to heterologous serotypes (conditional on having a PRNT50 < 40 to the homologous serotype) and the odds of becoming ill in the absence of immunity to any serotype.

The odds ratio of clinical case illness in children with low antibody titres (PRNT50 < 40) to a specific serotype, conditional on different PRNT50 thresholds for the other serotypes (serving as indicators of previous exposure) can be found in Fig. 1. These results reinforce the serotype-specific interactions described above but also demonstrate that even low titres can have a disease enhancing effect and highlight the non-linear relationship nature of serotype interactions. We have highlighted in red, the serotype interactions identified as significant in the multinomial logistic model in Fig. 1 and observe how the odds ratios of clinical disease are modulated by imposing different PRNT50 cut-offs for heterologous immunity. The classification that considers PRNT50 = 40 as the homologous immunity threshold for both homologous and heterologous titres is the most consistent with the multinomial logistic regression. We present the Odds Ratios for different titre cut-off values in Supplementary Fig. S4.

**Figure 2:**
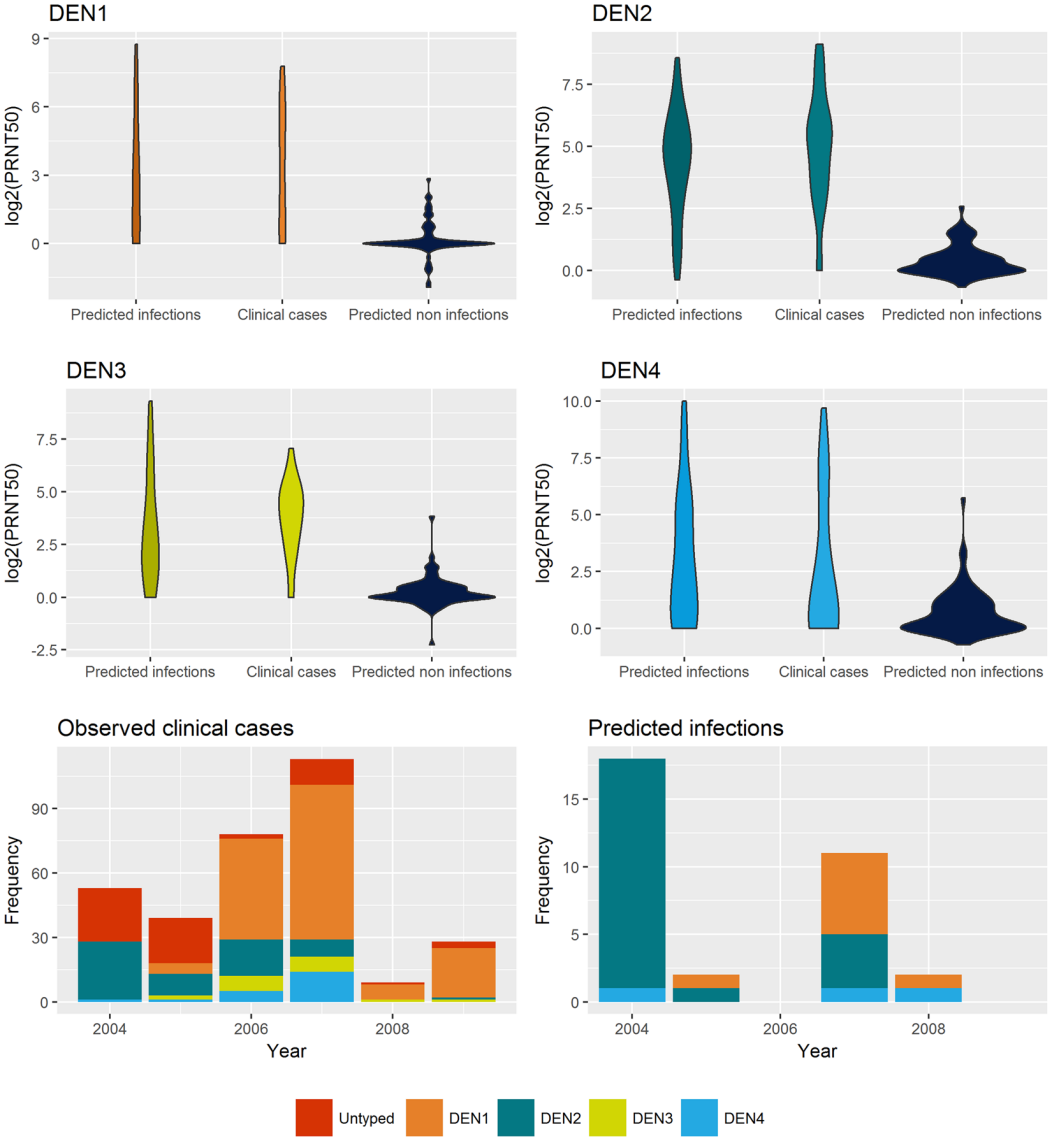
Evaluation of the asymptomatic infection prediction model. The violin plots in the top two rows display the distribution of the mean rise in titres (across all serotypes) in consecutive years for predicted infections and predicted non-infections compared to the observed cases. The bottom row shows the time series of predicted infections by serotype (right) compared to the observed dengue clinical case time series (left).

The overall best fitting model reveals a positive age dependence on the odds of developing clinical dengue DENV2 illness, suggesting older children are at higher risk of DENV2 disease. Even more significant is the effect of calendar year, which clearly shows a temporal increase in the odds of DENV2 illness along with a decrease in DENV1 clinical case odds. This is in line with the observed serotype replacement pattern observed in Figs 2 and S1.

The best fitting model including IgG and IgM as covariates (model 87) suggest that elevated IgG titres are disease enhancing (positive coefficients), whereas increased IGM titres do not seem to affect the likelihood of having dengue illness (coefficient are very close to zero).

Noteworthy is the inclusion of a covariate on the breadth of immune repertoire in the logistic model with the highest log-likelihood (but second highest AIC). This covariate is a binomial response variable which indicates whether there are at least two measured PRNT50 titres over 40 per sample. Interestingly, the estimated coefficients suggest a difference between having a raised antibody against a single serotype compared to having a broader antibody repertoire, with no enhancement in the latter but rather a cross-protection effect. Considering antibody repertoires with multiple PRNT50 titres over 80 does not improve the fit. We investigate this relationship further by calculating the Odds Ratios of clinical disease given combinations of homologous and heterologous PRNT50 titres, much like in Fig. 1. The results (Supplementary Fig. S3) indicate that, in this study, disease enhancement is more likely in children with pre-existing elevated antibody titres against a single serotype.

### PRNT50 titres as indicators of asymptomatic infections

Using our longitudinal dataset, we implemented a random forest algorithm (RFA) to predict the likely outcomes in individuals whose antibody titres (to any of the serotypes) rose between any two consecutive years. The non-serotype specific RFA trained on individuals with dropping or unchanged titres together with those that were clinical cases had a predictive error of 0.55%, only misclassifying 2 in 363 data entries. A total of 37 asymptomatic dengue infections were predicted to have occurred in the 109 children displaying some immunity boost. The antibody rises observed in these children are similar to those observed in clinical cases, and markedly different from the ones observed in individuals predicted to not have been infected (Fig. 2). The serotype specific RFA performed similarly well, with a 1.4% prediction error, although it was unable to discriminate infections with DENV3 and DENV4, due to the very low number of cases with these serotypes in the training set (2 and 1 respectively). DENV1 and DENV2 cases were accurately predicted in 6 out of 7 and 7 out of 8 children. Also, 4 out of 5 untyped clinical cases were predicted to be DENV2 infections, whereas the remaining case showed no antibody rise in titres in the year following illness and was thus predicted to not have been the result of a dengue infection. The time-varying serotype-specific incidence of predicted infections (comprising a set of the 4 untyped clinical cases and 29 asymptomatic infections) displays the same DENV2 to DENV1 serotype replacement pattern seen in clinical cases (bottom right panel in Fig. 2).

Estimation of these asymptomatic infections in a small subset of individuals with sequential PRNT50 measurements allowed us to extrapolate the proportion of infected individuals in the whole cohort and to thus get an estimate for the total number of sub-clinical infections in the population and ultimately the asymptomatic infection to clinical case ratio, which shows substantial fluctuation over time (Supplementary Fig. S5).

## Discussion

Dengue pathogenesis is extremely complex. Following a dengue infection, individuals enter a cross-serotype immunity period of unknown duration followed by life-long immunity from homologous challenges along with what can only be described as a controversial and multi-factorial heterologous risk enhancement. Here we show that baseline immunity to any serotype (defined as having any detectable titre) offers a substantive reduction in dengue illness risk against any serotype (RR = 0.81 [0.67–0.99]) while higher titres offer an increased benefit (Supplementary Table S1). Indeed, there has been some evidence in the literature for heterologous neutralization with antibodies targeting DENV protein epitopes that are shared among serotypes^3,49^.

Given our knowledge of the immune profile of children at the start of a given year and of their clinical manifestations throughout the year, we have partitioned our population into a range of immune subgroups defined by their PRNT50 titres. For each serotype, the subgroup with undetectable titres to any serotype was defined as the control group and odds ratios of dengue illness were calculated for each other subgroup (Table 2). We found that the odds of clinical occurrence are significantly enhanced in children with low-to-intermediate (PRNT50 < 40) homologous titres combined with intermediate to high (PRNT50 > 40) heterologous titres, consistent with reported antibody dependent enhancement effects^22,32,33,38^. These results are observed across all serotypes but not as significant for DENV1, which shows an exceedingly competent homologous neutralizing potential, with titres over 40 inducing protection regardless of the heterologous context. Lower protective neutralising titres for DENV1 compared to other serotypes were also described in^50^. For all serotypes, high antibody titres to the homologous and at least one of the heterologous serotypes induce clinical protection upon infection with any serotype. Due to the low numbers of individuals in some of the subgroups (see Tables S5–S8), many relative risks are not statistically significant. However, the observed trends are consistent across all serotypes. We must stress that lack of significance does not necessarily imply a relationship does not exist as there might be insufficient statistical power in the data and to ascertain any serotype interaction. The numbers in the top left corner of cells in Table 2 indicates the subset of PRNT50 titre bins where a statistically significant interaction could potentially be detected (mostly limited to low homologous titres) – in this case, those with a grade of evidence equal to 3. The odds ratio for DENV2 (38.06) albeit statistically significant should be carefully interpreted, as it results from a very small number of cases. Both viral neutralization and ADE effects have been associated with the number of antibodies bound to the virion^51,52^. ADE has been extensively studied *in vitro* and occurs when antibody levels are lower than the threshold for neutralization, as long as there is sufficient number of antibodies to support stable attachment of the virion to cells^53^. Here we further characterise this hypothesis, showing an enhancement effect for homologous antibody titres under the protective threshold (here found to be PRNT50 = 40) and heterologous antibody titres over said threshold. These results are evident when exploring the odds ratios for different PRNT titre cut-offs to define immunity status as shown in Fig. 1, and fit with the non-linearity previously shown in^3,38^. These studies established that pre-existing antibody titres against any serotype are key in determining disease risk and what shape that risk regulating immunity function takes. Whilst Salje *et al*.^3^ show the existence of a post infection stable antibody setpoint of 1 year, after which a PRNT titre threshold of 40 is deemed to be protective, Katzelnik and colleagues^38^ clearly demonstrate a serotype non-specific ADE effect in humans. Here, we retrieve both results and add a critical component to that narrative by identifying serotype-specific pairs that are linked to increased clinical dengue risk.

We must stress that in this study we can only correlate clinical outcomes with measured PRNT50 titres from sera collected in the 12 months prior to clinical illness, thus operating on a short time scale and blinded to long term antibody titre dynamics. The one year setpoint followed by exponential decay suggested in^3^ offers assurances that high antibody titres are a reflection of recent infections, thus validating our time frame. We cannot, however, make inferences on the cumulative nature of exposure. The data analysed in this study also contain a small subset of children which were followed up for 2 years or more, allowing for a dengue disease outcome prediction model to be constructed. We used this subset of children for which we have measured PRNT50 titres in consecutive years as training data for a random forest algorithm that predicts putative clinical outcomes. We developed both a serotype-specific and non-specific version of this algorithm reaching a better predictive performance than standard multinomial logistic regression models for prediction of the infecting dengue serotypes^54^. Out of 217 individuals (some of which with multiple data entries) 24 children fell ill during the follow up period. Our RFA proposes 37 more infections (33 for which the infecting serotype is predicted as well) to have occurred, resulting in an apparent to symptomatic case rate ranging from 1.88 to 11.72 in accordance with has been described for other trials sites in SE Asia^22,55,56^, and predicted by a similar method^3^.

These results paint a picture of extremely complex cross-serotype interactions underpinning dengue epidemiology. Multinomial models can help disentangle some of these properties and generalise cross-serotype interactions, possibly shedding some light on the relevance of particular combinations of sequential infections. Our multinomial logistic regression model offers a moderate predictive power as evidenced by the AUC values in Supplementary Table S4. This is not surprising given that this was not a controlled challenge study and an unknown subset of the non-cases was likely not exposed to any dengue virus. The purpose of the multinomial logistic model is to explore how the immunity landscape of the population affects the probability of specific outcomes, thus unveiling cross-serotype interactions. It does so to great effect, showing clear signals of increased risk of clinical DENV2 illness due to elevated anti-DENV1 antibodies and to a lesser extent by increased IgG levels. High anti-DENV1 titres also seem to increase the occurrence of DENV4 cases and decrease the chances of DENV3 illness. Indeed, the sequences of infections which we find to translate into a higher risk of clinical dengue illness upon secondary infection are: DENV1 followed by DENV2, DENV1 followed by DENV4, DENV2 followed by DENV3 and DENV4 followed by DENV3. Some of these sequences have been fairly well documented in other settings, particularly the increased risk of DENV2 following DENV1 infection^32,37,57^. Plotting titre data as a function of the resulting clinical dengue serotype (Supplementary Fig. S2) clearly illustrates that a considerable number of children falling ill with DENV2 or DENV4 previously had high titres to DENV1. Conversely, low anti-DENV1 titres are found in individuals latter succumbing to DENV1 or DENV3 illness.

Phylogenetics of the dengue virus suggests that it clusters into two genetic clusters: one with DENV1 and DENV3, and another containing DENV2 and DENV4^58^. This genetic clustering seems to have a direct translation to an antigenic relationship, with DENV1 and DENV3 displaying significant cross-protection, with anti-DENV antibodies raised against one of these viruses suggested to protect against disease caused by the other (Fig. 1 and Table 3). Strikingly, in this study we find risk enhancement interactions exclusively between serotypes in different genetic clusters. The absence of a significant DENV1 enhancement by DENV2 antibodies is also striking in the context of the observed serotype replacement dynamics in Vietnam during this period (Fig. 2) and suggests that the DENV2 replacement by DENV1 was not driven by antibody dependent enhancement but was rather the result of an epidemiological context characterised by a generalised lack of immunity to DENV1.

Although throughout this study we have only made links between humoral immunity and the risk of dengue clinical illness, the role of other factors, namely that of cellular mediated immunity, cannot be understated. Whilst antibody responses, when taken in isolation from other components of the immune system, can modulate the severity of *in vivo* infections, the presence of cellular immunity can nullify any enhancement effect^59^, or on the contrary contribute to severe disease^27^. Also in murine models, it has been reported that lethal antibody enhancement of dengue disease can be prevented by Fc modification^60^. More recently, severe dengue illness was explained by a substantial production of IgGs with enhanced affinity for the activating Fc receptor FcγRIIIA^61^, whilst another study found higher dengue IgG levels in symptomatic infections compared to asymptomatic ones^62^. Thus, the coefficients retrieved for IgG here might be an indication that children displaying increased IgG tires had high affinity IgGs. In this study we report serotype specific interactions leading to dengue illness enhancement, particularly highlighting sequences of infections (conditional on certain PRNT50 titre thresholds being met) with different serotypes which lead to a statistically significant increase in disease risk. We also reinforce the potential role of IgGs in inducing dengue illness. The results presented are a mere reflection of the observable phenomenon of enhancement and its association with pre-infection antibody titres and not a mechanistic or causal explanation of the role played by antibodies in the disease enhancement effect.

This study further highlights the extreme complexity of dengue pathogenesis. It uncovers disease enhancement effects across specific serotype pairs, suggesting the most likely sequences of infection in dengue secondary clinical cases. Whilst we appreciate that the implementation of serological screening cannot be performed routinely in large populations can be challenging, our results offer some guidance as to how severe future epidemics could potentially be, given the serotype replacement history in an epidemiological setting. Furthermore, our results suggest that vaccines that confer broadly neutralizing antibody responses could alleviate enhancement related concerns, something that became clear from the CYD-TDV vaccine efficacy analyses^46^.

## Supplementary information

Supplementary information

## Data Availability

Qualified researchers may request access to patient level data and related study documents including the clinical study report, study protocol with any amendments, blank case report form, statistical analysis plan, and dataset specifications. Patient level data will be anonymized and study documents will be redacted to protect the privacy of trial participants. Further details on Sanofi’s data sharing criteria, eligible studies, and process for requesting access can be found at: https://www.clinicalstudydatarequest.com.
