# Supplementary material for: Cross-serotype interactions and disease outcome prediction of dengue infections in Vietnam
Source: Sci Rep. 2019 Jun 28;9:9395. doi: 10.1038/s41598-019-45816-6 (PMC6598999; doi:10.1038/s41598-019-45816-6)
Supplement: Supplementary file 1 — Supplementary information [file 41598_2019_45816_MOESM1_ESM.pdf]

Supplementary information for:

Cross-serotype interactions and disease outcome prediction of dengue infections in Vietnam

Authors: R. Aguas[1,3,4], I. Dorigatti[1], L.Coudeville[2], C. Luxemburger[2], N.M. Ferguson[1]

***Author affiliation*:**

[1]MRC Centre for Global Infectious Disease Analysis, School of Public Health, Imperial College London, Faculty of Medicine, Norfolk Place, London, W2 1PG, UK.

[2]Sanofi Pasteur, 14 espace Henry Vallée, 69007 Lyon,France

[3]Centre for Tropical Medicine and Global Health, Nuffield Department of Medicine, University of Oxford, Oxford, United Kingdom

[4]Mahidol-Oxford Tropical Medicine Research Unit, Faculty of Tropical Medicine, Mahidol University, Bangkok, Thailand

Corresponding author: r.aguas@imperial.ac.uk

Supplementary Figure S1. Epidemiological data collected over the study period. The panels on the left show the age profiles of dengue case occurrences during the study period by gender and serotype. The right most panels display clinical dengue illness time series by gender and serotype.

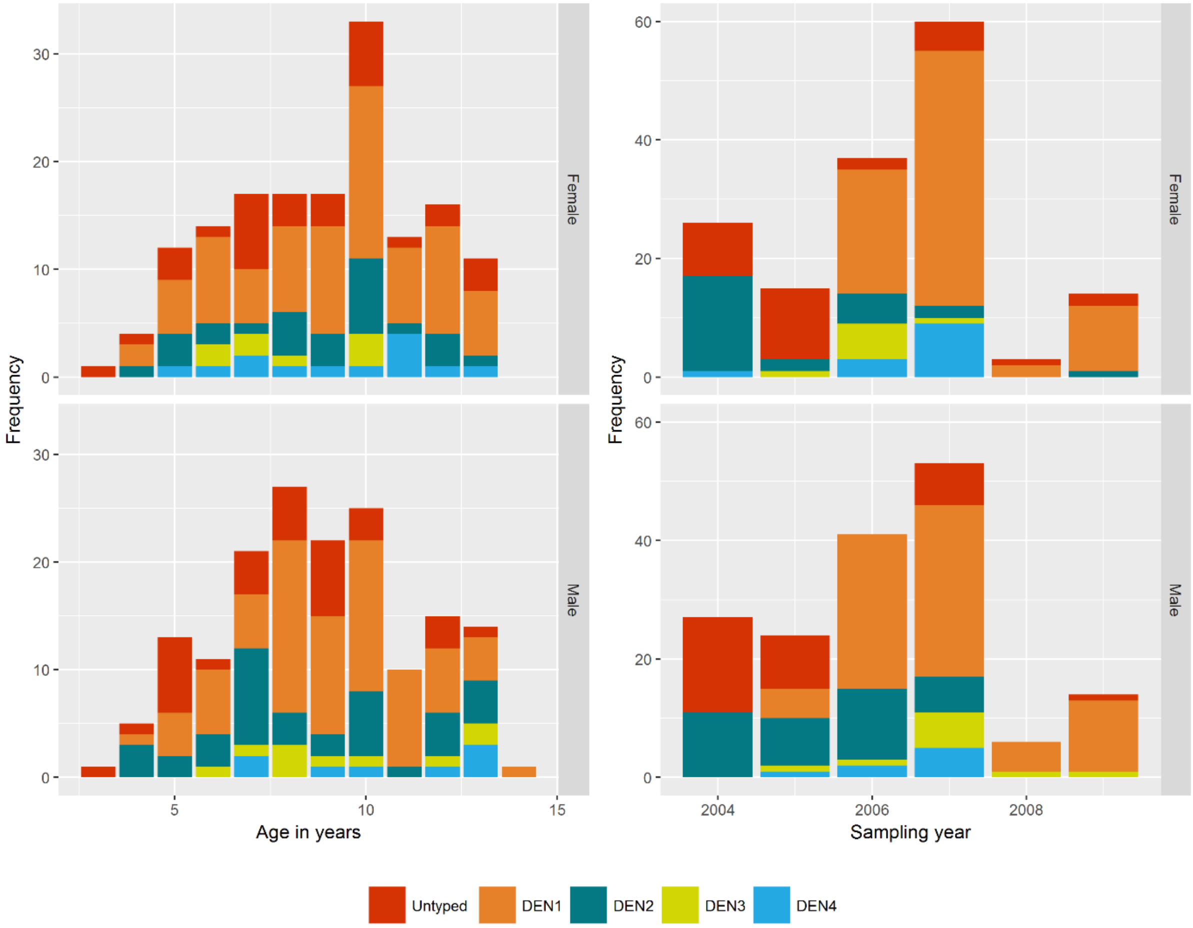

Supplementary Figure S2. Measured antibody profiles of dengue cases. The radar plot on the left shows the mean anti-DENV titres measured in children with dengue fever. Each line is coloured according to the infecting dengue serotype. The violin plots on the right show the distribution of anti-DENV antibodies in clinical cases for each infecting serotype. Each panel refers to the measured PRNT50 titres against a specific serotype.

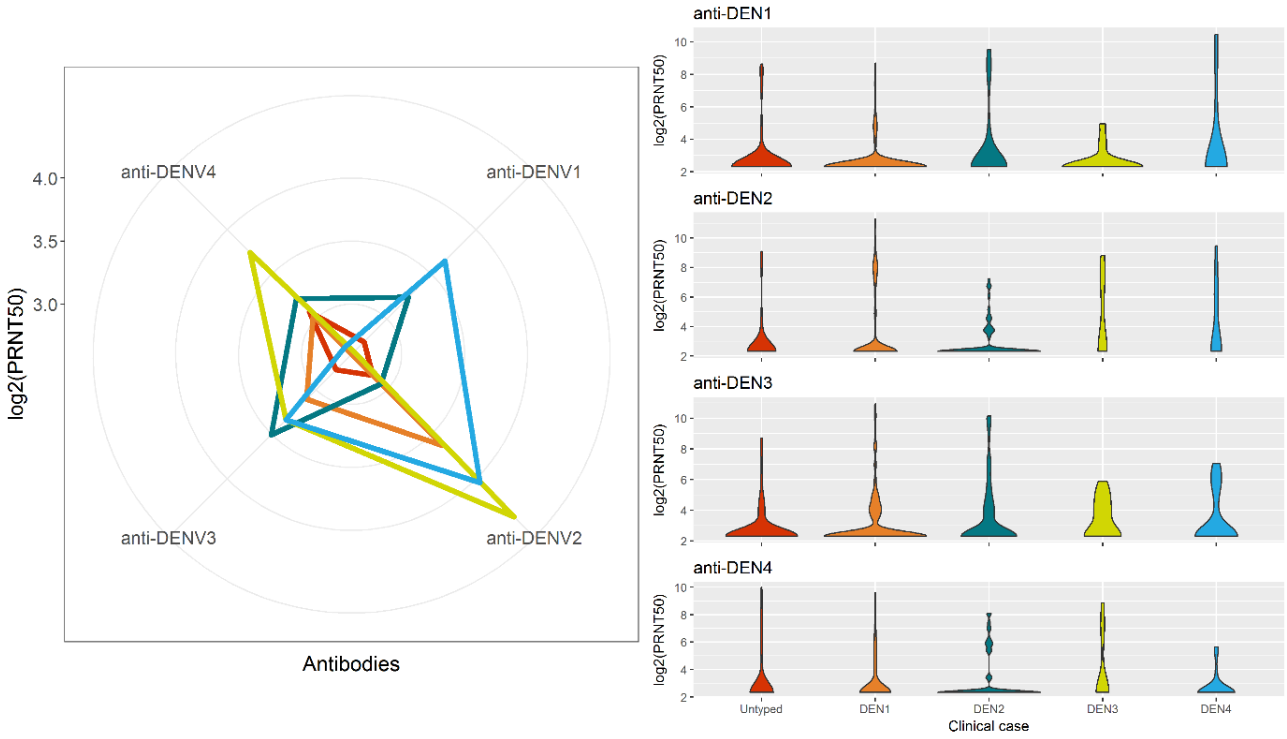

Supplementary Figure S3. Odds ratio of dengue illness in the year following neutralising antibody titre measurements in children with different antibody repertoires. The top row of plots refers to children with monotypic immunity, i.e., only one antibody over PRNT50=40, while the bottom row is for children with at least two PRNT titres over 40.

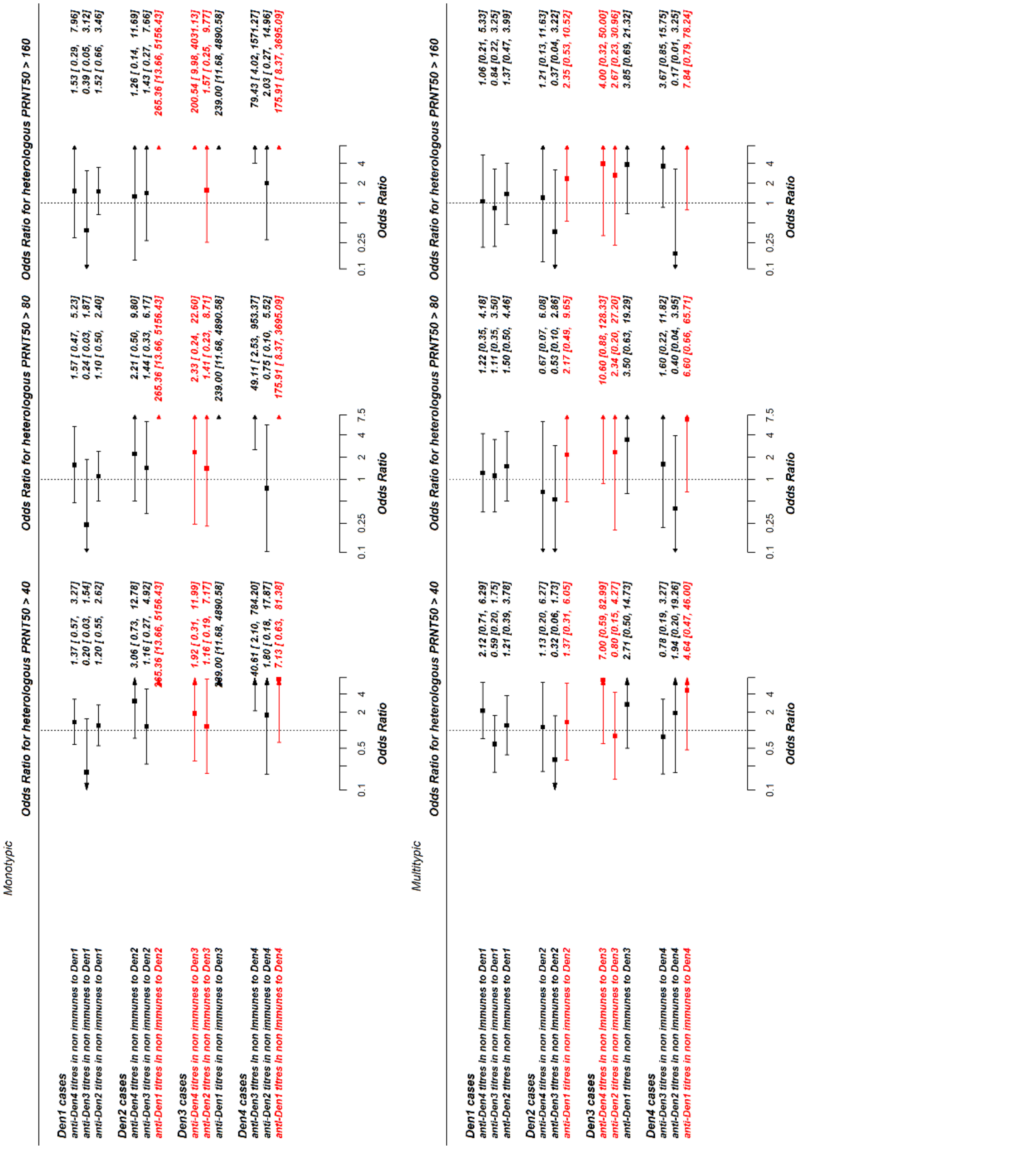

Supplementary Figure S4. The odds ratio presented here refers to children with a homologous PRNT50 titre under 40, i.e., non-immune to the reference dengue serotype thus reflecting the ratio between the odds of having clinical dengue illness when immune to heterologous serotypes (conditional on having a PRNT50<40 to the homologous serotype) and the odds of becoming ill in the absence of immunity to any serotype.

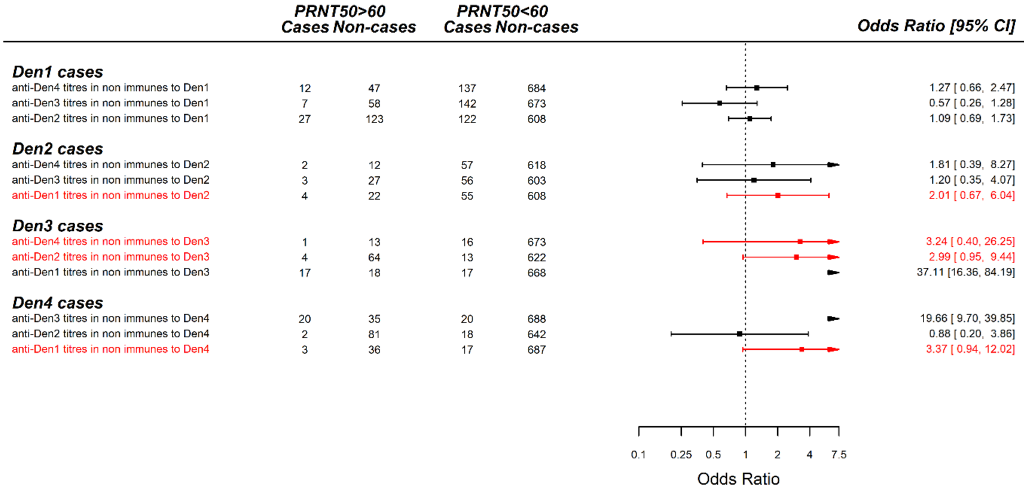

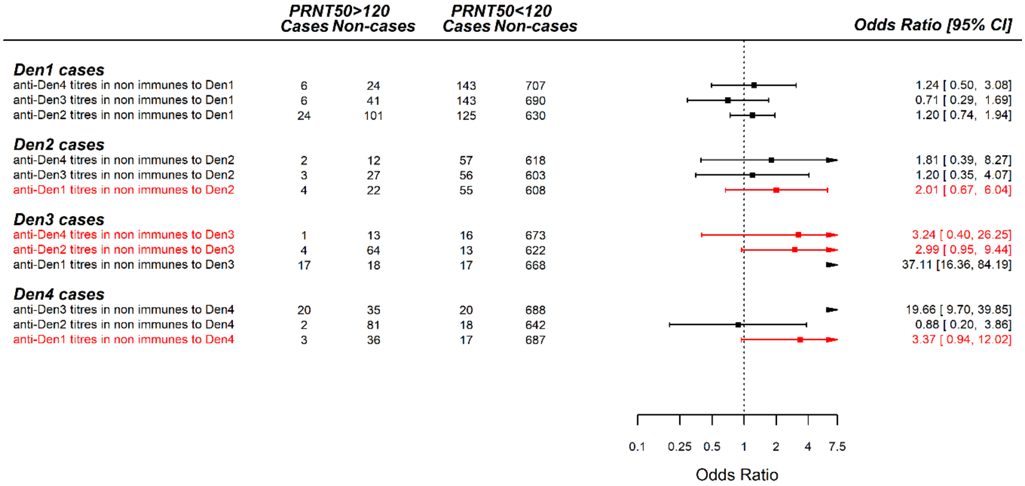

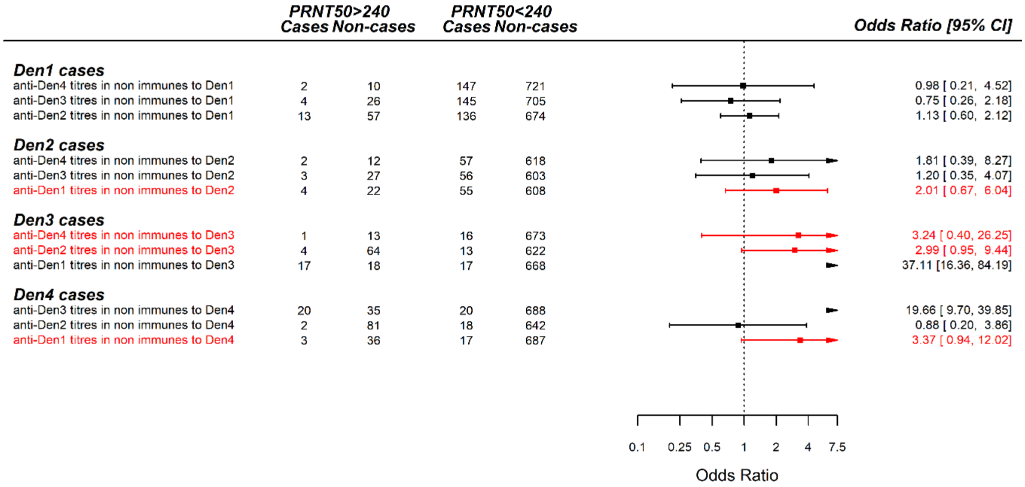

Supplementary Figure S5. Predicted asymptomatic infections in the study cohort and estimated asymptomatic infection to clinical case ratios throughout the years. The yearly figures are presented as stacked lines on a logarithmic scale to highlight the temporal dynamics. We could not predict asymptomatic infections in 2006 due to the low number of PRNT50 measurements in non-clinical individuals.

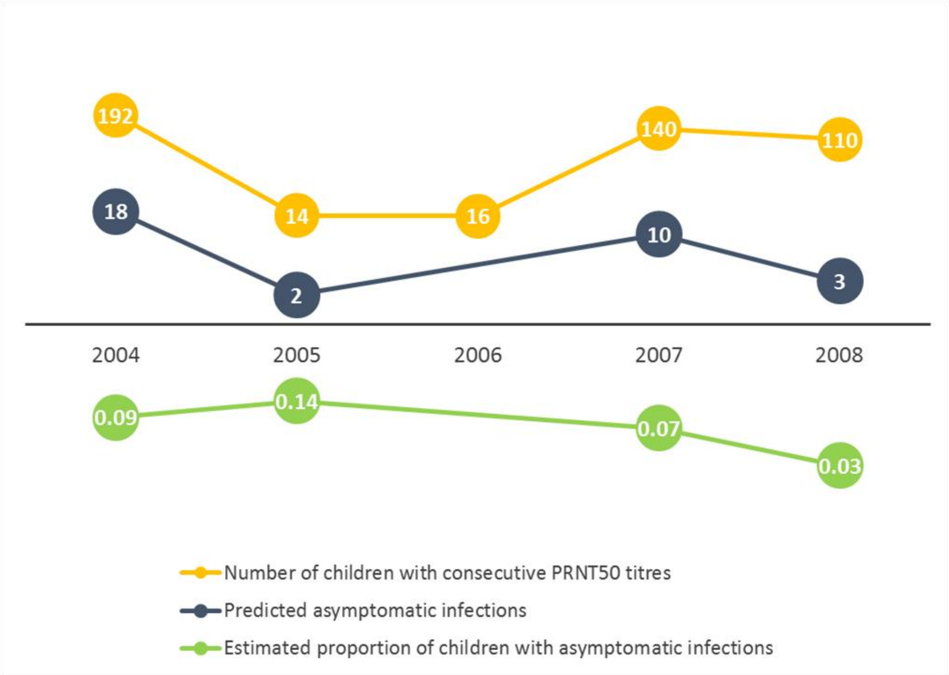

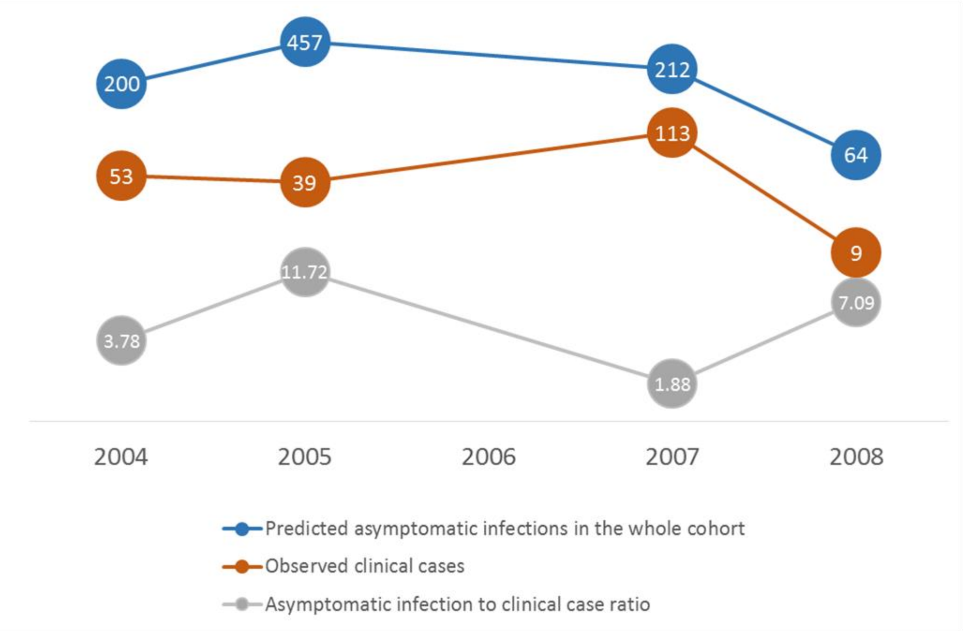

Supplementary Table S1. Odds ratio of clinical dengue illness depending on multiple immunity cut-offs. BIS stands for baseline immune status, as defined in each scenario.

Immune defined as having any PRNT50 titre >=10		
	**Immune**	**Naive**
Cases	117	203
Non cases	374	488
BIS OR	0.752 (0.577-0.979)	
p-value	**0.0346**	

Immune defined as having any PRNT50 titre >= 40		
	**Immune**	**Naive**
Cases	102	218
Non cases	343	519
BIS OR	0.708(0.540-0.929)	
p-value	**0.0128**	

Immune defined as having any PRNT50 titre >= 80		
	**Immune**	**Naive**
Cases	83	237
Non cases	304	558
BIS OR	0.643(0.483-0.856)	
p-value	**0.0025**	

Immune defined as having any PRNT50 titre >= 160		
	**Immune**	**Naive**
Cases	66	254
Non cases	254	608
BIS OR	0.622 (0.457-0.846)	
p-value	**0.0025**	

Supplementary Table S2. Variables used in the multinomial regression models.

Name	Variable description
Age	Age in years (continuous)
Gender	Male or Female (binary)
X_i	PRNT50 titres for each serotype (Log2 dilutions)
Year	Calendar year
BIS	Baseline immune status (any PRNT50 value over detectability limit = 1, else = 0)
BIS80	Baseline immune status (any PRNT50 value over 80 =1, else = 0)
Multi	Multitypic baseline immune profile (over detection threshold for more than one serotype =1, else =0)
Multi40	Multitypic baseline immune profile (PRNT50>40 for more than one serotype =1, else =0
Multi80	Multitypic baseline immune profile (PRNT50>80 for more than one serotype =1, else =0
Skewedness	Skewedness of the concatenated vector of PRNT50 titres for all serotypes. Indicates the breadth (or lack thereof) of the antibody repertoire.
Dominance	Immune dominance indicator (if there is one PRNT50 value greater than twice the sum of all others =1, else 0)
P_i	Immune status against each serotype (binary – 1 if over detection limit, 0 otherwise)
$P80_i$	Immune status against each serotype (binary – 1 if PRNT50>80, 0 otherwise)
IgG	IGG titre measured by an ELISA test
IgM	IGM titre measured by an ELISA test
IgGp	Positive IGG test (binary- 1 if GOD/cut-off>1.1, 0 otherwise)
IgMp	Positive IGM test (binary- 1 if GOD/cut-off>1.1, 0 otherwise)

Supplementary Table S3. Multinomial logistic regression models explored.

Variable	Model																						
	1	2	3	4	5	6	7	8	9	10	11	12	13	14	15	16	17	18	19	20	21	22	23
Age			x	x			x						x	x									
Gender		x		x			x																
X_i	x	x	x	x	x	x	x	x								x	x	x		x			
Year																							
BIS					x				x	x				x	x								
BIS80						x	x				x	x	x								x		
Multi								x		x		x	x	x								x	x
Multi40																	x						
Multi80																		x					
Skewedness																						x	
Dominance															x	x					x		
P_i																			x	x			x
$P80_i$																							
IGG																							
IGM																							
IGGp																							
IGMp																							
**Variable**	**Model**																						
	24	25	26	27	28	29	30	31	32	33	34	35	36	37	38	39	40	41	42	43	44	45	46
Age				x			x											x			x		x
Gender					x																		x
X_i			x	x	x	x	x								x	x	x	x	x	x	x	x	
Year																							
BIS							x																
BIS80																							
Multi				x	x																		
Multi40	x		x			x		x									x	x	x	x	x		
Multi80									x														
Skewedness						x																	
Dominance			x																x				
P_i	x																						
$P80_i$		x																					
IGG										x				x	x		x	x	x	x	x	x	
IGM											x			x	x		x	x	x				
IGGp												x				x							
IGMp													x			x							
**Variable**	**Model**																						
	47	48	49	50	51	52	53	54	55	56	57	58	59	60	61	62	63	64	65	66	67	68	69
Age			x	x			x						x	x									
Gender		x		x			x																
X_i	x	x	x	x	x	x	x	x								x	x	x		x			
Year	x	x	x	x	x	x	x	x	x	x	x	x	x	x	x	x	x	x	x	x	x	x	x
BIS					x				x	x				x	x								
BIS80						x	x				x	x	x								x		
Multi								x		x		x	x	x								x	x
Multi40																	x						
Multi80																		x					
Skewedness																						x	
Dominance															x	x					x		
P_i																			x	x			x
$P80_i$																							
IGG																							
IGM																							
IGGp																							

IGMp																							
**Variable**	**Model**																						
	70	71	72	73	74	75	76	77	78	79	80	81	82	83	84	85	86	87	88	89	90	91	92
Age				x			x											x			x		X
Gender					x																		X
X_i			x	x	x	x	X								x	x	x	x	x	x	x	x	
Year	X	X	x	x	x	x	x	X	X	X	X	X	X	X	x	x	x	x	x	x	x	x	X
BIS							x																
BIS80																							
Multi				x	x																		
Multi40	x		x			x		x									x	x	x	x	x		
Multi80									x														
Skewedness						x																	
Dominance			x																x				
P_i	x																						
$P80_i$		x																					
IGG										x				x	x		x	x	x	x	x	x	
IGM											x			x	x		x	x	x				
IGGp												x				x							
IGMp													x			x							

Supplementary Table S4. Model evaluation and selection. We underline models with AIC less than 4 AIC units away from the minimum AIC value.

Model	AIC	Number of parameters	AUC1	AUC2	AUC3	AUC4
**1**	1706.5	20	0.590	0.623	0.694	0.644
**2**	1711.4	24	0.587	0.650	0.683	0.688
**3**	1708.9	24	0.613	0.618	0.677	0.697
**4**	1713.8	28	0.611	0.654	0.673	0.727
**5**	1711.7	24	0.587	0.650	0.683	0.688
**6**	1713.4	24	0.590	0.623	0.693	0.655
**7**	1713.4	24	0.590	0.623	0.693	0.655
**8**	1709.3	24	0.597	0.653	0.692	0.659
**9**	1750.5	8	0.528	0.515	0.538	0.502
**10**	1752.5	12	0.552	0.522	0.570	0.533
**11**	1746.1	8	0.541	0.536	0.555	0.502
**12**	1751.6	12	0.538	0.549	0.551	0.500
**13**	1753.6	16	0.565	0.559	0.566	0.603
**14**	1755.0	16	0.577	0.552	0.575	0.607
**15**	1753.9	12	0.533	0.516	0.588	0.563
**16**	1714.0	24	0.591	0.611	0.694	0.643
**17**	1707.1	24	0.595	0.625	0.690	0.643
**18**	1710.8	24	0.589	0.611	0.675	0.647
**19**	1742.3	20	0.597	0.566	0.597	0.615
**20**	1718.7	36	0.609	0.663	0.731	0.648
**21**	1748.1	12	0.549	0.541	0.576	0.555
**22**	1747.9	12	0.561	0.504	0.550	0.539
**23**	1751.6	12	0.531	0.510	0.578	0.569
**24**	1739.3	12	0.548	0.545	0.572	0.532
**25**	1710.3	20	0.576	0.606	0.704	0.604
**26**	1713.8	28	0.595	0.628	0.691	0.665
**27**	1711.1	28	0.617	0.670	0.677	0.706
**28**	1714.4	28	0.593	0.667	0.682	0.682
**29**	1712.7	28	0.592	0.626	0.674	0.633
**30**	1713.9	28	0.613	0.651	0.695	0.701
**31**	1733.5	8	0.551	0.544	0.510	0.522
**32**	1735.5	8	0.543	0.531	0.502	0.506
**33**	1747.3	8	0.551	0.589	0.666	0.661
**34**	1745.1	8	0.506	0.549	0.639	0.607
**35**	1747.0	8	0.530	0.540	0.543	0.522
**36**	1751.3	8	0.500	0.504	0.504	0.504
**37**	1750.0	12	0.513	0.582	0.641	0.623
**38**	1706.7	28	0.623	0.647	0.733	0.748
**39**	1715.4	28	0.605	0.638	0.709	0.662
**40**	1706.2	32	0.621	0.682	0.729	0.763
**41**	1707.4	36	0.624	0.669	0.734	0.772
**42**	1712.8	36	0.623	0.676	0.729	0.766
**43**	1704.6	28	0.620	0.684	0.744	0.768
**44**	1705.8	32	0.622	0.669	0.737	0.749
**45**	1704.6	24	0.623	0.647	0.749	0.742
**46**	1753.8	12	0.526	0.553	0.528	0.627
47	***1643.8***	24	0.702	0.755	0.749	0.672
**48**	1648.4	28	0.705	0.760	0.751	0.700
49	***1639.9***	28	0.711	0.765	0.741	0.691
**50**	1644.1	32	0.711	0.770	0.738	0.723
**51**	1649.9	28	0.705	0.760	0.751	0.700

**52**	1650.8	28	0.707	0.755	0.749	0.696
**53**	1650.8	28	0.707	0.755	0.749	0.696
**54**	1647.9	28	0.703	0.754	0.744	0.701
**55**	1693.5	12	0.661	0.712	0.578	0.546
**56**	1694.7	16	0.685	0.716	0.594	0.625
**57**	1689.4	12	0.668	0.706	0.580	0.553
**58**	1693.8	16	0.680	0.713	0.586	0.575
**59**	1688.0	20	0.670	0.728	0.608	0.612
**60**	1688.9	20	0.671	0.728	0.621	0.617
**61**	1695.9	16	0.666	0.712	0.612	0.625
**62**	1651.4	28	0.704	0.757	0.750	0.670
**63**	1646.2	28	0.705	0.755	0.747	0.711
**64**	1649.2	28	0.702	0.755	0.754	0.674
**65**	1683.9	24	0.701	0.715	0.650	0.665
**66**	1662.1	40	0.717	0.763	0.759	0.703
**67**	1691.2	16	0.677	0.712	0.601	0.618
**68**	1688.7	16	0.688	0.713	0.590	0.592
**69**	1692.2	16	0.674	0.713	0.608	0.612
**70**	1681.7	16	0.689	0.714	0.599	0.571
**71**	1653.9	24	0.706	0.735	0.726	0.657
**72**	1653.1	32	0.704	0.754	0.745	0.705
**73**	1644.9	32	0.711	0.768	0.734	0.704
**74**	1652.8	32	0.706	0.760	0.743	0.702
**75**	1653.5	32	0.706	0.756	0.756	0.699
**76**	1646.8	32	0.711	0.767	0.747	0.696
**77**	1676.6	12	0.687	0.711	0.557	0.560
**78**	1677.5	12	0.684	0.709	0.547	0.574
**79**	1694.6	12	0.641	0.719	0.599	0.642
**80**	1692.6	12	0.631	0.711	0.666	0.647
**81**	1695.4	12	0.648	0.713	0.565	0.550
**82**	1698.0	12	0.636	0.710	0.553	0.545
**83**	1697.2	16	0.632	0.719	0.673	0.649
**84**	1644.8	32	0.715	0.756	0.774	0.762
**85**	1654.1	32	0.706	0.758	0.742	0.702
**86**	1646.8	36	0.713	0.758	0.777	0.751
87	***1642.1***	40	0.716	0.772	0.790	0.752
**88**	1653.7	40	0.712	0.759	0.776	0.759
**89**	1645.2	32	0.711	0.761	0.759	0.756
90	***1642.0***	36	0.716	0.769	0.744	0.725
91	***1642.8***	28	0.715	0.756	0.760	0.737
**92**	1688.7	16	0.654	0.732	0.602	0.638

Supplementary Table S5. Number of subjects in each immunity subgroup used for odds ratio calculations with serotype 1 as a reference.

Serotype 1

Cases

		Heterologous titres					
		<10	[10,20[	[20,40[	[40,80[	[80,160[	≥160
Homologous titre	<10	96	6	4	7	7	20
	[10,20[	0	0	0	0	1	2
	[20,40[	0	0	0	0	2	4
	[40,80[	0	0	0	0	0	2
	[80,160[	0	6	0	0	0	1
	≥160	0	0	1	0	0	1

Non cases

		Heterologous titres					
		<10	[10,20[	[20,40[	[40,80[	[80,160[	≥160
Homologous titre	<10	488	10	15	34	41	77
	[10,20[	0	0	0	1	5	25
	[20,40[	0	1	4	1	3	25
	[40,80[	0	0	2	1	0	24
	[80,160[	0	0	1	1	0	15
	≥160	0	5	11	6	9	57

Supplementary Table S6. Number of subjects in each immunity subgroup used for odds ratio calculations with serotype 2 as a reference.

Serotype 2

Cases							
		Heterologous titres					
		<10	[10,20[	[20,40[	[40,80[	[80,160[	≥160
Homologous titre	<10	38	2	0	2	2	3
	[10,20[	0	1	0	2	2	3
	[20,40[	0	0	0	1	0	3
	[40,80[	0	0	0	0	0	1
	[80,160[	0	0	0	0	0	3
	≥160	0	0	0	0	0	0

Non cases							
		Heterologous titres					
		<10	[10,20[	[20,40[	[40,80[	[80,160[	≥160
Homologous titre	<10	488	6	12	21	10	15
	[10,20[	4	0	2	5	5	22
	[20,40[	4	0	2	4	6	24
	[40,80[	6	0	3	0	1	25
	[80,160[	17	10	2	0	0	15
	≥160	12	25	25	13	8	70

Supplementary Table S7. Number of subjects in each immunity subgroup used for odds ratio calculations with serotype 3 as a reference.

Serotype 3

		Heterologous titres					
**Cases**		<10	[10,20[	[20,40[	[40,80[	[80,160[	≥160
Homologous titre	<10	9	0	0	0	1	0
	[10,20[	0	0	0	1	1	2
	[20,40[	0	0	0	0	0	3
	[40,80[	0	0	0	0	0	1
	[80,160[	0	0	0	0	0	0
	≥160	0	0	0	0	0	0
**Non cases**		<10	[10,20[	[20,40[	[40,80[	[80,160[	≥160
Homologous titre	<10	488	9	11	21	19	17
	[10,20[	0	1	5	8	15	32
	[20,40[	0	1	4	4	6	45
	[40,80[	3	0	2	1	1	27
	[80,160[	3	3	4	0	0	27
	≥160	3	6	14	12	7	64

Supplementary Table S8. Number of subjects in each immunity subgroup used for odds ratio calculations with serotype 4 as a reference.

Serotype 4

Cases

		Heterologous titres					
		<10	[10,20[	[20,40[	[40,80[	[80,160[	≥160
Homologous titre	<10	12	0	0	2	0	4
	[10,20[	0	0	0	0	0	1
	[20,40[	0	0	0	0	1	0
	[40,80[	0	0	0	0	0	1
	[80,160[	0	0	0	0	0	0
	≥160	0	0	0	0	0	0

Non cases

		Heterologous titres					
		<10	[10,20[	[20,40[	[40,80[	[80,160[	≥160
Homologous titre	<10	488	4	8	15	39	86
	[10,20[	5	1	1	1	1	40
	[20,40[	7	4	0	1	1	20
	[40,80[	14	6	2	0	0	17
	[80,160[	1	5	3	1	0	12
	≥160	5	1	6	3	1	63

Supplementary Table S9. Odds ratio of clinical dengue illness depending on multiple homologous immunity cut-offs per serotype. BIS stands for baseline immune status, as defined in each scenario, and BIS OR is then the Odds Ratio of clinical dengue with each serotype in individuals with antibody tires above a certain threshold compared with the Odds Ratio of children with lower antibody titres.

DENV1			***DENV2***			***DENV3***			***DENV4***		
Immune defined as having any PRNT50 titre >=10											
	Immune	***Naive***		***Immune***	***Naive***		***Immune***	***Naive***		***Immune***	***Naive***
Cases	*20*	*133*	*Cases*	*16*	*47*	*Cases*	*8*	*10*	*Cases*	*3*	*18*
Non cases	*197*	*665*	*Non cases*	*310*	*552*	*Non cases*	*298*	*565*	*Non cases*	*222*	*640*
BIS OR	*0.508 (0.309-0.834)*		*BIS OR*	*0.606 (0.338-1.087)*		*BIS OR*	*1.517 (0.592-3.884)*		*BIS OR*	*0.4805 (0.140-1.647)*	
p-value	***0.0074***		*p-value*	***0.0930***		*p-value*	***0.3852***		*p-value*	***0.2435***	
Immune defined as having any PRNT50 titre >= 40											
	Immune	***Naive***		***Immune***	***Naive***		***Immune***	***Naive***		***Immune***	***Naive***
Cases	*11*	*140*	*Cases*	*4*	*59*	*Cases*	*1*	*17*	*Cases*	*1*	*20*
Non cases	*132*	*730*	*Non cases*	*232*	*630*	*Non cases*	*177*	*686*	*Non cases*	*134*	*722*
BIS OR	*0.708 (0.216-0.777)*		*BIS OR*	*0.1841 (0.066-0.512)*		*BIS OR*	*0.228 (0.030-1.725)*		*BIS OR*	*0.269 (0.036-2.024)*	
p-value	***0.0063***		*p-value*	***0.0012***		*p-value*	***0.1521***		*p-value*	***0.2025***	
Immune defined as having any PRNT50 titre >= 80											
	Immune	***Naive***		***Immune***	***Naive***		***Immune***	***Naive***		***Immune***	***Naive***
Cases	*9*	*142*	*Cases*	*3*	*60*	*Cases*	*0*	*18*	*Cases*	*0*	*21*
Non cases	*105*	*757*	*Non cases*	*197*	*665*	*Non cases*	*143*	*720*	*Non cases*	*95*	*761*
BIS OR	*0.457 (0.226-0.924)*		*BIS OR*	*0.169 (0.052-0.544)*		*BIS OR*	*0.136 (0.008-2.265)*		*BIS OR*	*0.185 (0.011-3.086)*	
p-value	***0.0292***		*p-value*	***0.0029***		*p-value*	***0.1643***		*p-value*	***0.2402***	
Immune defined as having any PRNT50 titre >= 160											
	Immune	***Naive***		***Immune***	***Naive***		***Immune***	***Naive***		***Immune***	***Naive***
Cases	*2*	*149*	*Cases*	*0*	*63*	*Cases*	*0*	*18*	*Cases*	*0*	*21*
Non cases	*88*	*774*	*Non cases*	*153*	*709*	*Non cases*	*106*	*757*	*Non cases*	*73*	*783*
BIS OR	*0.118 (0.029-0.485)*		*BIS OR*	*0.0364 (0.002-0.592)*		*BIS OR*	*0.192 (0.011-3.213)*		*BIS OR*	*0.248 0.015-4.134)*	
p-value	***0.0025***		*p-value*	***0.0199***		*p-value*	***0.2511***		*p-value*	***0.3313***	
